# Influence of Secondary Electromagnetic Stirring and Soft Reduction on Slab Macrosegregation Evolution of E355 Steel

**DOI:** 10.3390/ma19061164

**Published:** 2026-03-17

**Authors:** Xin Xie, Peng Shi, Baohui Yuan, Chenhui Wu, Daiwei Liu

**Affiliations:** 1 Pangang Group Research Institute Co., Ltd., Panzhihua 617000, China15808102178@163.com (P.S.); yuanbaohui20@163.com (B.Y.); wch_neu@126.com (C.W.); 2 School of Electrical and Information Engineering, Panzhihua University, Panzhihua 617000, China

**Keywords:** slab, electromagnetic stirring, soft reduction, banded structure

## Abstract

Macrosegregation in continuous casting slabs remains a critical defect that adversely affects the homogeneity and mechanical properties of the final rolled products. Industrial experiments were conducted on E355 steel continuous casting slabs to investigate the effects of electromagnetic stirring (EMS) and soft reduction (SR) on the evolution of slab macrosegregation. Furthermore, the inheritance of segregation from the slab to the rolled plate was analyzed. The results indicate that the equiaxed crystal ratio increases and the centerline segregation decreases with increasing stirring intensity. The application of both secondary EMS and SR minimized the centerline segregation in the slab. When the current intensity was increased from 0 A to 320 A in continuous stirring mode, the equiaxed crystal fraction increased from 22.52% to 32.52%, and the centerline segregation index decreased from 1.23 to 1.17. Compared with the continuous stirring mode, the alternating stirring mode promoted a more pronounced increase in the equiaxed crystal ratio and a further reduction in the centerline segregation. The centerline segregation in the slab correlates with the banded structure observed in the rolled plate. A higher degree of slab centerline segregation corresponds to a more severe banded structure and greater fluctuations in the mechanical properties of the plate. Through parameter optimization, the recommended settings are an alternating stirring mode with a current of 320 A at 5 Hz and an SR amount of 3 mm. Under these optimized conditions, the equiaxed crystal ratio of the slab increased to 35.22%, the centerline segregation index dropped to 1.15, and the banded structure in the rolled plate was reduced to grade 2.0. Consequently, the standard deviations of the tensile strength and elongation were 8.03 MPa and 1.1%, respectively.

## 1. Introduction

During solidification, solute is continuously rejected from the solid phase and enriched in the liquid phase due to solute partitioning between the two phases [1,2]. As solidification proceeds, macrosegregation forms in the core region of the strand. This macrosegregation cannot be eliminated by subsequent heating and rolling processes, leading to significant fluctuations in the mechanical properties of the rolled plate [3,4]. In severe cases, it can lead to the scrapping of the final product. Consequently, the formation of macrosegregation in continuous casting strands, its inheritance as banded structures in rolled products, and the resultant variations in mechanical properties have been extensively studied for several decades.

Suzuki et al. [5] investigated the effect of EMS on the solidification of ingots. They found that a weak stirring intensity combined with an alternating stirring mode could effectively alleviate V-segregation in ingots. Their work also suggested that ingots should solidify with an equiaxed crystal structure, and that the width of the liquid cavity should be maintained between 30 and 50 mm. Ayata et al. [6] analyzed the influence of combined EMS on the centerline segregation of high-carbon steel strands. They reported that mold EMS could produce a wider equiaxed zone and a region of negative segregation with reduced severity. They noted that when the solid fraction is high or the stirring current is low, the flow fails to sufficiently penetrate the two-phase (mushy) zone. However, shrinkage porosity tends to form under conditions of either a high solid fraction or a low stirring intensity. Xu et al. [7] studied the effect of EMS on internal cracks in 1Cr13 stainless steel. They found that EMS effectively reduced solute segregation when the liquid cavity width and stirring current were 50 mm and 250 A, respectively. However, near the end of solidification, the application of EMS was found to promote the formation of white bands. Liu et al. [8] simulated fluid flow and solidification behavior in a strand subjected to secondary cooling EMS for E355 steel. The alternating stirring mode was more advantageous, as it promoted a more uniform flow field, reduced variations in the solidification end point, and thus mitigated carbon segregation. The average carbon segregation index was reduced to 1.227 using alternating stirring at 320 A and 5 Hz. Using a coupled multiphase solidification model, Jiang [9] analyzed the evolution of the solidification structure and centerline segregation under EMS for Q345 steel. The electromagnetic force drives the flow of liquid steel in the mushy zone, thereby promoting solute transport and heat transfer during solidification. However, the centerline segregation index in the strand was only reduced to 1.26.

By applying a certain amount of reduction near the solidification end of the strand, the solute-enriched liquid in the mushy zone can be prevented from concentrating at the center, thereby eliminating centerline segregation and porosity. Bleck et al. [10] applied soft reduction (SR) during ingot solidification and found that centerline segregation in bearing steel 100Cr6 could be alleviated when the central solid fraction was between 0.89 and 0.94. However, the effect of SR became negligible when the central solid fraction exceeded 0.96. Qi et al. [11] performed a soft reduction test on a slab continuous caster. They determined that the optimal central solid fraction and reduction amount ranges were 0.3–0.9 and 5.7–7.2 mm, respectively. Furthermore, they reported that a larger reduction amount was required to mitigate sulfur (S) segregation compared to carbon (C) segregation. Xu et al. [12] conducted experiments involving heavy reduction during the continuous casting of wide-thick Q370R steel slabs. They observed that the solidification endpoint was located between 1/4 and 1/8 of the slab width, where the segregation index reached 1.4. When a 9 mm reduction was applied with a central solid fraction ranging from 0.53 to 0.98, the centerline segregation index was significantly reduced to 1.1. By coupling a thermodynamic model with a solute segregation model, Zhao et al. [13] analyzed the effects of bulge deformation and soft reduction on solute distribution in 40Cr steel slabs. Their results showed that when the solid fraction in the reduction zone was below 0.8 or 0.86, the centerline segregation index was significantly reduced, reaching as low as 1.005. Furthermore, they found that SR significantly reduced central porosity when the solid fraction was high.

Once macrosegregation forms in the strand, it is difficult to eliminate during subsequent heating and rolling processes. Su [14] reported that chromium (Cr) segregation between dendrites was significant and diffused slowly during the heat treatment process. Even after treatment at 1260 °C for 60 min, the carbides did not fully dissolve. Therefore, reducing solute segregation in the continuous casting strand is key to improving the banded structure in the final rolled plate. Ji et al. [15] analyzed the correlation between segregation defects in the slab and banded structures in the rolled plate. They found that the concentrations of carbon (C), chromium (Cr), molybdenum (Mo), and manganese (Mn) were elevated at segregation spots within the equiaxed zone. They concluded that reducing both mold EMS intensity and the equiaxed crystal ratio could mitigate spot segregation and, consequently, improve the banded structure in the rolled plate. An et al. [16] demonstrated that the severity of the banded structure in rolled GCr15 steel decreased with a lower segregation index. Their work showed that applying EMS and SR could effectively reduce the centerline segregation index to between 0.96 and 1.08. Jiang et al. [17] studied the inheritance of segregation from slab to plate in E355 steel. They observed that during heating and rolling, segregation spots smaller than 30 μm completely dissolved, whereas larger spots persisted and formed banded structures. In addition, the slow diffusion rate of manganese (Mn) was identified as a key factor in banded structure formation.

Previous research has extensively utilized numerical simulations and laboratory experiments to investigate the effects of EMS [5,6,7,8,9] and SR [10,11,12,13] on the evolution of macrosegregation in slabs. The inheritance of segregation and its influence on banded structure formation have also been analyzed [14,15,16,17]. However, most studies have been based on simulations and laboratory experiments [5,8,9,10,11,14,15,16,17,18], with only a limited number conducted at an industrial scale [6,7,11,12]. In this work, industrial experiments were conducted to investigate the effects of EMS and SR on the solidification macrostructure and centerline segregation evolution. Furthermore, the relationships among slab segregation, banded structure, and the mechanical properties of the rolled material were revealed through macro-etching and mechanical testing.

## 2. Research Scheme and Methodology

**Figure 1:**
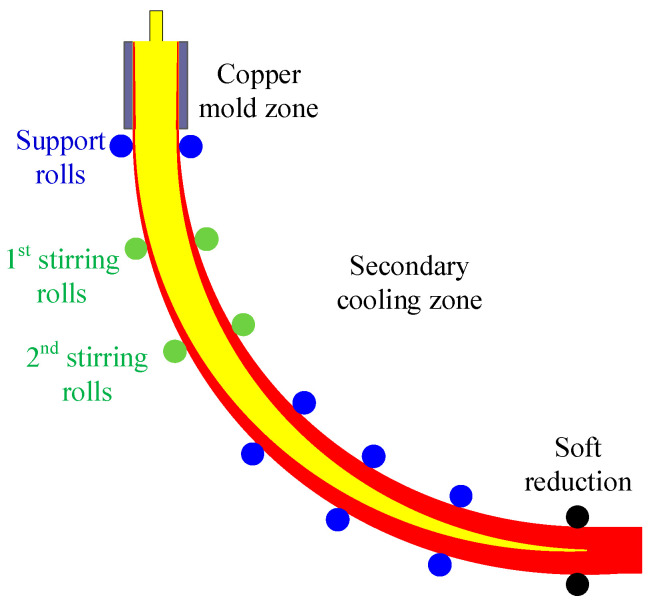
Schematic of continuous casting process.

In the slab continuous casting process, high-temperature molten steel is fed into the mold through a submerged entry nozzle. The copper mold extracts heat, causing the molten steel temperature to drop continuously and a solidified shell to form. The strand then enters the secondary cooling zone, where it gradually solidifies further under the spray water cooling. To enhance the internal quality of the strand, secondary electromagnetic stirring (EMS) and soft reduction (SR) technologies are widely employed, as illustrated in Figure 1. In the industrial caster, the first and second pairs of stirring rollers are located 3.0 m and 4.7 m from the meniscus, respectively. The rollers can operate in either continuous or alternating stirring modes, thereby driving fluid flow within the two-phase (mushy) zone. Soft reduction is applied near the solidification end of the strand. The reduction zone spans from 17 to 21 m from the meniscus, with a total reduction amount of 3 mm. This process squeezes the solute-enriched melt within the two-phase region, thereby reducing segregation defects in the strand.

To analyze the influence of EMS and SR on the internal quality of the slab, eight distinct experimental schemes were designed and implemented, as detailed in Table 1. Schemes 1 to 4 were conducted under a continuous stirring mode to investigate the effect of stirring current intensity on the solidification structure and segregation. Similarly, Schemes 5 to 7 employed an alternating stirring mode to study the effect of varying stirring currents on internal quality. In Scheme 8, SR was omitted. A comparison between Scheme 7 (with SR) and Scheme 8 (without SR) was made to isolate and analyze the specific impact of SR on slab internal quality. The chemical composition of the investigated E355 steel (in wt.%) was C: 0.17, Si: 0.23, Mn: 1.4. The casting speed and superheat were maintained in the ranges of 1.15–1.25 m/min and 20–30 °C, respectively.

**Figure 2:**
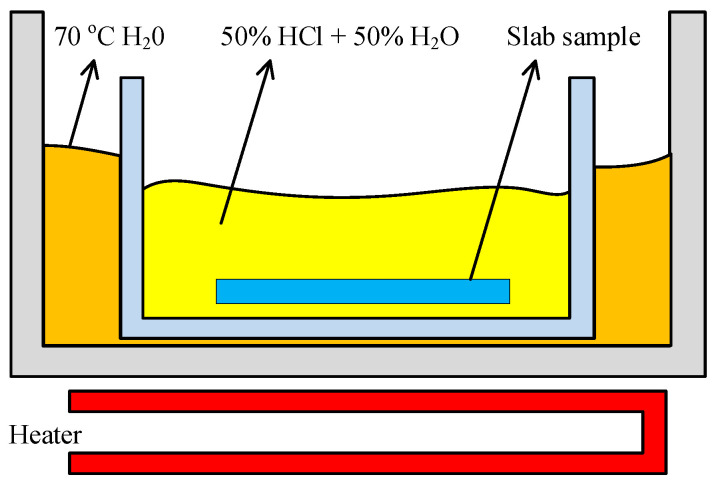
Schematic of sample hot-acid corrosion.

During the experiment, samples were extracted from both the slab and the subsequently rolled plate at the quarter-width position. The solidification macrostructure of the continuous casting slab was revealed by hot-acid etching, as shown in Figure 2. The slab samples were sectioned, polished, and then etched in a solution of 50 vol.% hydrochloric acid and 50 vol.% water at 70 °C for 10 min. This procedure revealed the slab’s macrostructure. Subsequently, to analyze the carbon distribution, drill samples were taken at 10 mm intervals along the transverse cross-section of the 230 mm thick slab. The segregation index (SI) for each sampling point was calculated using the equation SI = C_i_/C_tundish_. Here, C_i_ represents the carbon content of an individual drill sample, and C_tundish_ is the reference carbon content, measured from a sample obtained from the tundish during casting. An SI value greater than 1.0 indicates positive segregation, whereas a value less than 1.0 indicates negative segregation.

The tensile properties of the rolled plate were evaluated using a tensile testing machine. Furthermore, the influence of slab segregation on the banded structure and mechanical properties of the rolled plate was investigated. The findings from this work are intended to provide guidance for the production of high-quality steel.

## 3. Results and Discussion

Common methods for controlling macrosegregation in continuous casting strands include low-superheat casting, electromagnetic stirring (EMS), soft reduction (SR), and heavy reduction. In practice, superheat is typically maintained within a range of 15–25 °C, making low-superheat casting a standard practice. However, the application of heavy reduction is limited in conventional casters due to mechanical constraints and is therefore less common in industrial settings. Consequently, electromagnetic stirring (EMS) and soft reduction (SR) have become the most widely adopted techniques for mitigating strand macrosegregation. This work presents plant trial investigations focusing on the relationship between slab macrosegregation and the properties of the final rolled products.

The methods for controlling the macroscopic segregation of the strand include low superheat casting, electromagnetic stirring, soft reduction, and heavy reduction. During the continuous casting, the superheat is generally within the range of 15 to 25 °C, so the lower superheat casting is commonly applied. The compression capacity of conventional casters is limited, so heavy reductions are less commonly applied in the industry. Currently, electromagnetic stirring and soft reduction are commonly used to control strand macrosegregation. This work has conducted experiments in the plant trials, and the relationship between slab marcrosegregation and the properties of rolled products is analyzed.

### 3.1. Effect of Continuous Stirring Mode

**Figure 3:**
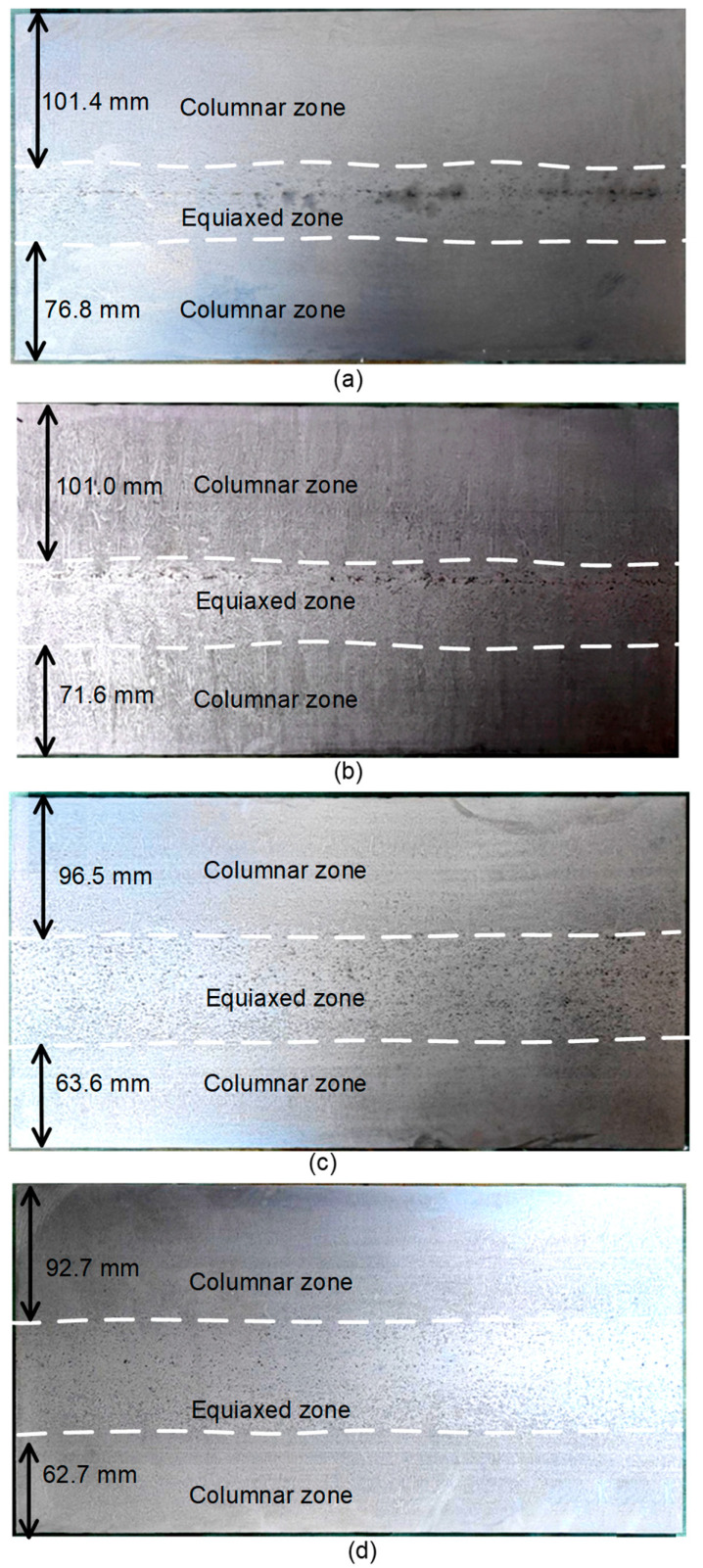
Etched macrostructure with different current intensities in the continuous stirring mode: (**a**) 0 A, (**b**) 160 A, (**c**) 240, and (**d**) 320 A.

Following polishing and hot-acid etching, the macrostructure of the slab cross-section was revealed. The distribution of the solidification structure is shown in Figure 3. Both the fixed and loose sides of the slab exhibited a columnar structure, while the core region comprised an equiaxed zone. With increasing EMS intensity, the length of the columnar zone decreased, and the equiaxed crystal ratio increased gradually. Concurrently, secondary EMS significantly improved the centerline segregation in the slab.

**Figure 4:**
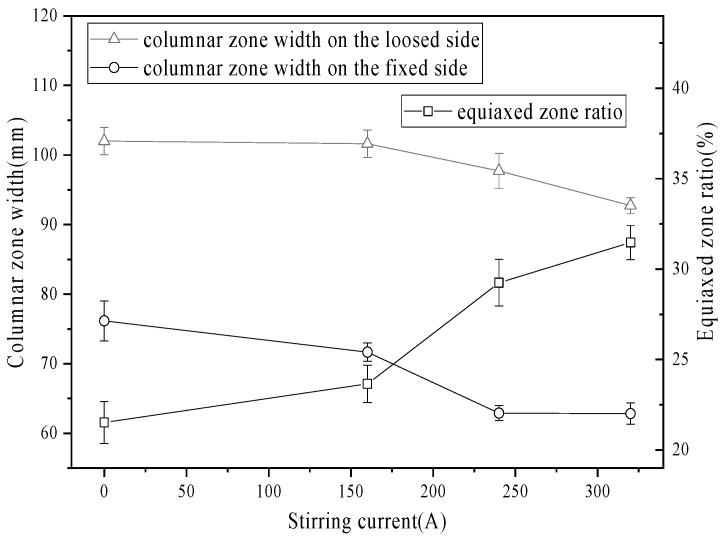
Columnar zone width and equiaxed zone fraction with different current intensities.

Figure 4 illustrates the widths of the columnar zone on the loose and fixed arc sides of the slab under the continuous stirring mode. The widths of the columnar zone exhibited a decreasing trend with increasing stirring current intensity. In the absence of EMS, the columnar zone widths were 101.4 mm on the loose side and 76.8 mm on the fixed side. When the stirring current was increased to 320 A, the corresponding widths decreased to 92.7 mm (loose side) and 62.7 mm (fixed side). The equiaxed crystal ratio increased with increasing current. As the current intensity was increased from 0 A to 320 A, the equiaxed zone ratio increased from 22.52% to 32.52%. This is attributed to the fluid flow induced by secondary EMS in the mushy zone, which fragments the columnar dendrites and enhances the nucleation rate [9].

**Figure 5:**
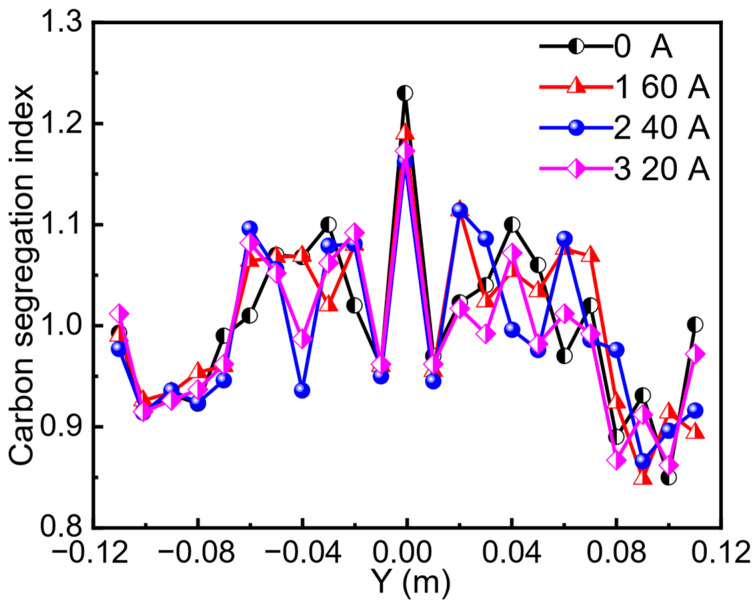
Carbon segregation index along the thickness with different current intensities.

The carbon segregation profiles along the slab thickness direction under different stirring current intensities are presented in Figure 5. Drill samples were taken along a line from the loose side to the fixed side, and their carbon content was analyzed using a carbon–sulfur analyzer. The results indicate that under all processing conditions, carbon exhibits a characteristic “W”-shaped distribution across the slab thickness. A pronounced negative segregation zone is evident approximately 50 mm beneath the slab surface. The solute (carbon) concentration increases with distance from the surface, leading to positive segregation in the central region of the slab. The maximum centerline segregation index decreased from 1.23 (without EMS) to 1.19, 1.16, and 1.17 with stirring currents of 160 A, 240 A, and 320 A, respectively. This indicates that the effect of EMS on further reducing segregation diminishes when the stirring current exceeds 240 A. This suggests that the promotion of solute homogenization by fluid stirring approaches a saturation point at around 240 A. Beyond this point (e.g., at 320 A), excessive stirring may occur, potentially remelting fragments and leading to a non-uniform distribution of solute, which can increase the carbon concentration at the center [8].

### 3.2. Effect of Alternative Stirring Mode

**Figure 6:**
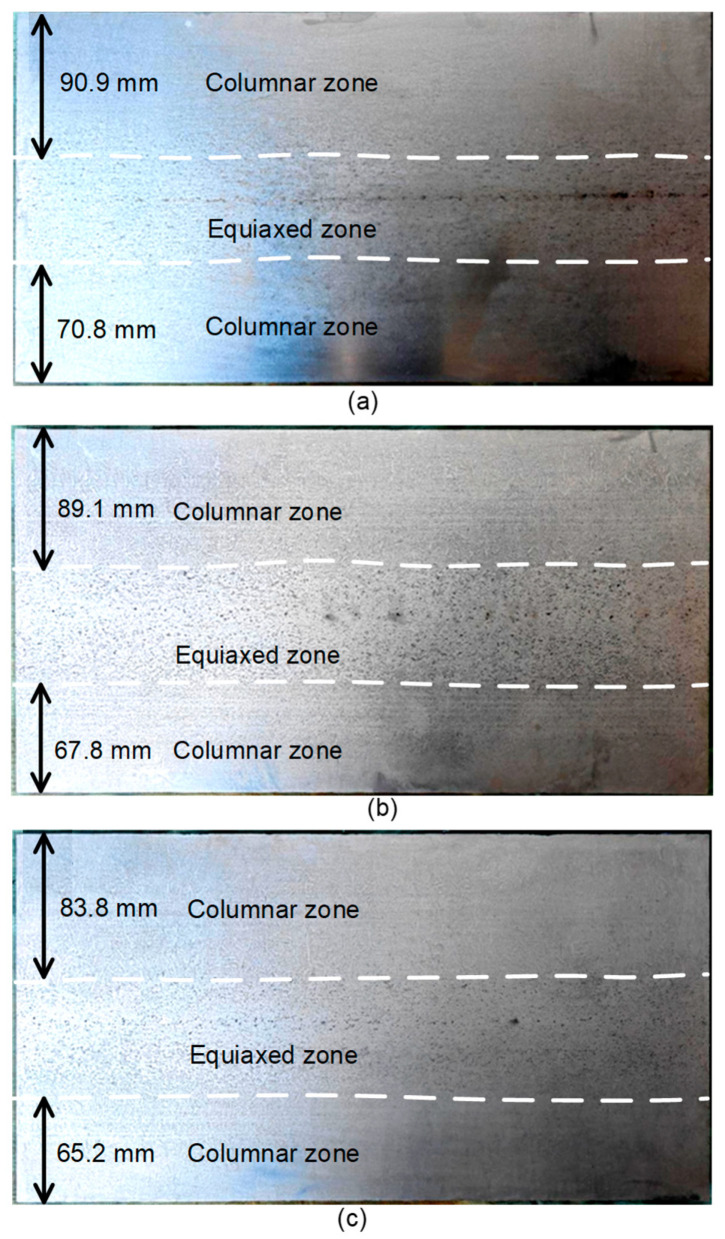
Etched macrostructure with different current intensities in the alternative stirring mode: (**a**) 160 A, (**b**) 240 A, and (**c**) 320 A.

The alternating stirring mode operates by periodically reversing the phase of the stirring current, thereby generating an alternating electromagnetic force. The current frequency was fixed at 5 Hz with an alternating period of 22 s, and current intensities of 160 A, 240 A, and 320 A were applied. Samples were obtained from industrially produced slabs and subsequently etched using the hot-acid method. The resulting macrostructures under different current intensities are shown in Figure 6. The alternating stirring mode effectively improved the centerline segregation of the slab. Furthermore, the degree of centerline segregation decreased significantly with increasing stirring intensity. Compared to continuous stirring at identical current levels, the periodic reversal of the Lorentz force in alternating mode more effectively fragments the tips of columnar dendrites. The resulting fragments are transported into a wider undercooled liquid region, where they act as nucleation sites. Simultaneously, the induced periodic flow enhances overall heat and mass transfer within the mushy zone. This promotes more uniform temperature and concentration fields, thereby reducing the thermodynamic driving force for the directional growth of columnar crystals [8].

**Figure 7:**
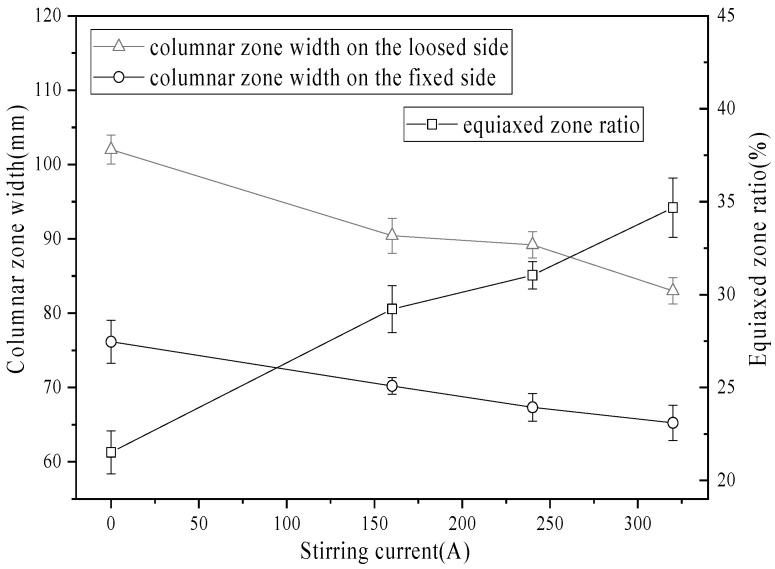
Columnar zone width and equiaxed zone ratio with different current intensities in the alternative stirring mode.

Figure 7 presents the widths of the columnar zones on the loose and fixed sides of the slab under alternating stirring conditions. The widths of the columnar zones on both sides decreased with increasing current intensity. Under alternating stirring with a current of 320 A, the columnar zone widths were reduced to 83.8 mm on the loose side and 65.2 mm on the fixed side. Consequently, the length of the columnar zone was significantly reduced. The equiaxed crystal ratio increased from 29.70% at 160 A to 35.22% at 320 A. Compared to the continuous stirring mode, the alternating mode yielded a higher equiaxed zone ratio. This enhancement is attributed to the wide-range fluid flow induced by alternating stirring, which promotes dendrite fragmentation and transports the fragments, thereby expanding the equiaxed zone.

**Figure 8:**
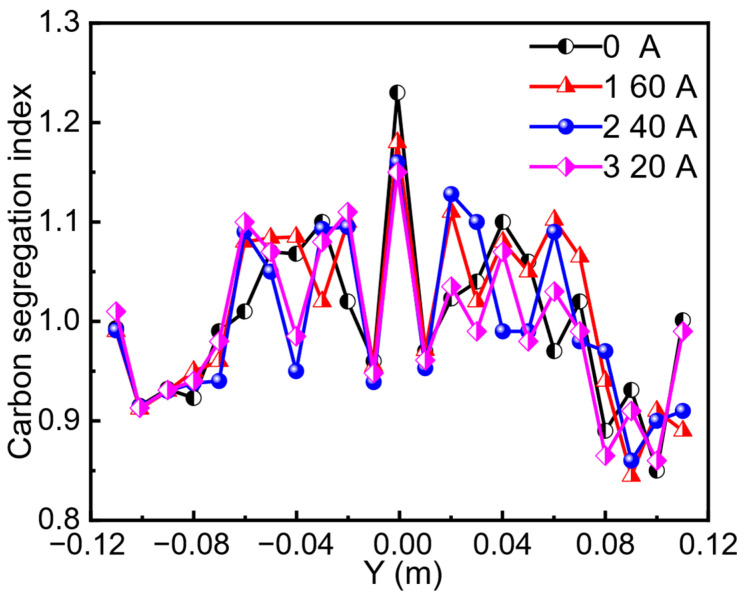
Carbon segregation index with different current intensities in the alternative stirring mode.

The segregation profile across the slab centerline under alternating stirring mode is presented in Figure 8. The segregation index decreased with increasing current intensity. Simultaneously, the range of the segregation index (the difference between maximum and minimum values) was reduced. At a current intensity of 160 A, the maximum segregation index and the segregation range were 1.18 and 0.33, respectively. When the current intensity was increased to 320 A, these values decreased to 1.15 and 0.29, respectively. Compared to continuous EMS, the alternating stirring mode resulted in slightly lower centerline segregation in the slab [8]. The periodically reversed electromagnetic force generated by alternating stirring continuously alters the flow direction within the mushy zone. This action breaks stable solute channels, promotes solute diffusion and mixing over a wider area, and more effectively fragments columnar dendrite tips, thereby increasing nucleation. Furthermore, this periodic flow may inhibit local solute over-enrichment, thereby mitigating the risk of “white band” defect formation.

### 3.3. Combined Effect of EMS and SR

**Figure 9:**
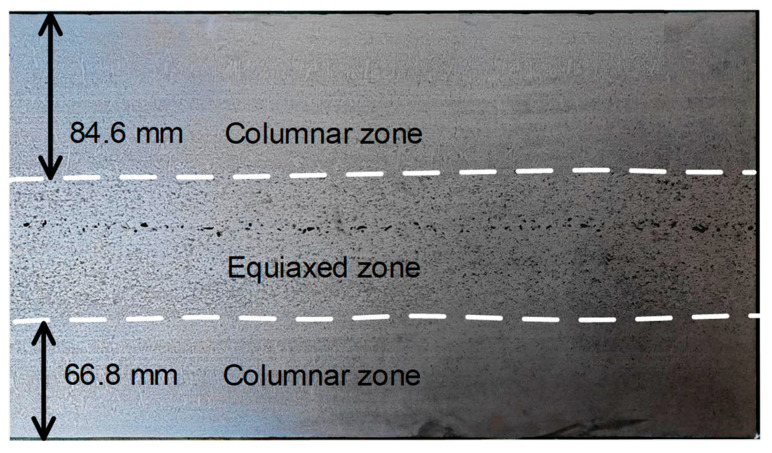
Etched macrostructure with S-EMS and without SR.

In continuous casting, EMS and SR are commonly employed to reduce centerline segregation in the slab, thereby improving the performance of the final rolled plate. To isolate the specific influence of SR on slab quality, SR was deliberately omitted in one strand while maintaining the alternating stirring mode. The alternating stirring mode was maintained in this strand, with a stirring current of 320 A at 5 Hz. The etched macrostructure of the slab sample from this strand is shown in Figure 9. The columnar zone widths were measured as 84.6 mm on the loose side and 66.8 mm on the fixed side. The equiaxed zone ratio in the slab core was 34.17%.

**Figure 10:**
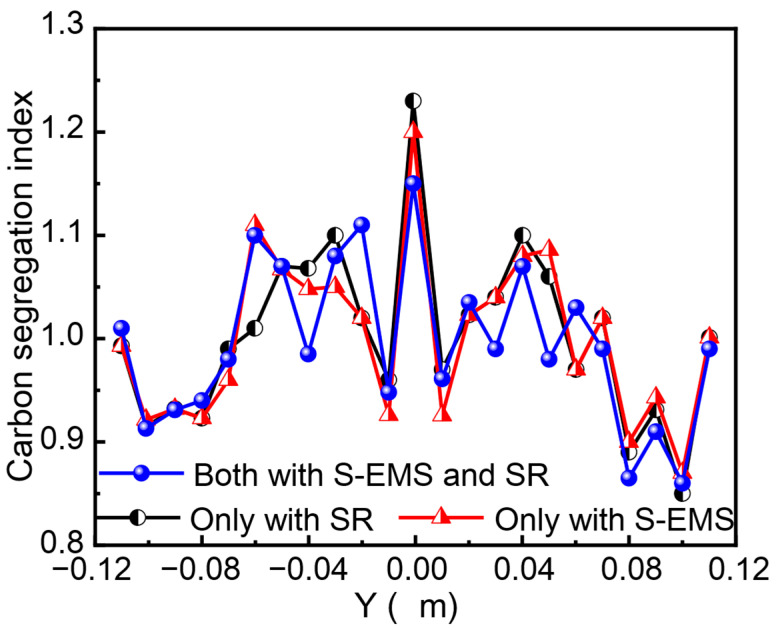
Carbon segregation index along the thickness with and without SR.

Figure 10 presents the carbon segregation profile along the centerline thickness direction under alternating EMS conditions, comparing cases with and without SR. Under the combined application of SR and EMS, the centerline segregation index of the slab was 1.15. In contrast, when only SR was applied (without EMS), the centerline segregation deteriorated, with the index increasing to 1.23. When only EMS was applied (without SR), the centerline segregation index was 1.20. Therefore, for industrial production, the combined application of EMS and SR is necessary to achieve a significant reduction in centerline segregation.

### 3.4. Heredity of Segregation from Slab to Rolled Plate

To further investigate the influence of slab segregation on the banded structure and mechanical properties of the rolled plate, slabs with different degrees of segregation were tracked through the rolling process. The rolled plates produced from slabs processed under three conditions—EMS only, SR only, and both EMS and SR—were studied.

**Figure 11:**
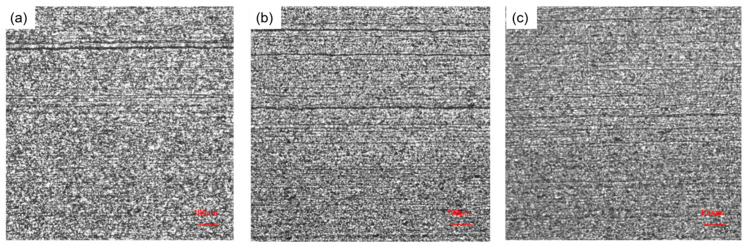
Distribution of banding structure in rolled material: (**a**) scheme 1, (**b**) scheme 8, and (**c**) scheme 7.

The slabs were heated to 1210 °C and hot-rolled to a final thickness of 2.1 mm. Samples were then taken from the plates, and the distribution and severity of the banded structure were characterized, as summarized in Table 2 and illustrated in Figure 11. For the slab processed with SR only, the centerline segregation index was 1.23, and the corresponding banded structure grade in the rolled plate was 5.0. For the EMS-only condition, the centerline segregation index and banded structure grade were 1.20 and 3.0, respectively. Under the combined EMS + SR condition, these values were further reduced to 1.15 and 2.0.

In the micrographs of Figure 11, the dark lines are pearlite bands. As the slab centerline segregation index decreased from 1.23 to 1.15, the maximum observed pearlite band width decreased correspondingly from 15 μm to 8 μm. Consequently, the microstructural homogeneity of the plate was improved. This confirms a direct correlation: a lower slab centerline segregation index results in a less severe banded structure in the rolled plate [17].

**Figure 12:**
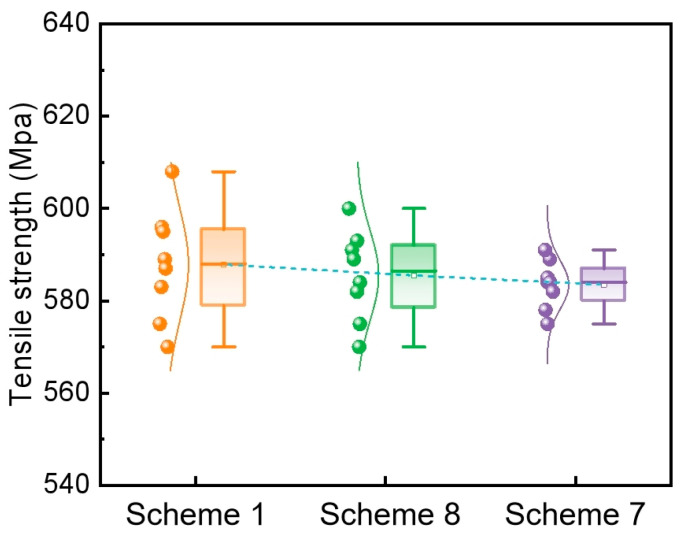
Distribution of tensile strength of rolled plate.

The tensile strength of the rolled plates was measured, with eight tests conducted for each experimental scheme, as shown in Figure 12. The average tensile strengths were 588 MPa for Scheme 1 (SR only), 586 MPa for Scheme 8 (EMS only), and 581 MPa for Scheme 7 (EMS + SR). Although a slight decreasing trend in average tensile strength is observed across the three schemes, all values meet the specified requirements. Scheme 1 exhibited the greatest fluctuation in tensile strength, whereas Scheme 7 showed the least.

The centerline positive segregation zones are locally enriched in carbon and manganese, which results in a locally higher volume fraction of pearlite upon cooling. Pearlite, particularly the cementite within it, is a strong and hard phase. Therefore, its increased local density contributes to the overall tensile strength. However, this microstructural heterogeneity simultaneously leads to mechanical property inhomogeneity, reflected in a larger standard deviation of tensile strength. Consequently, while more severe slab segregation correlates with a marginally higher average tensile strength, it also results in greater property fluctuations.

**Figure 13:**
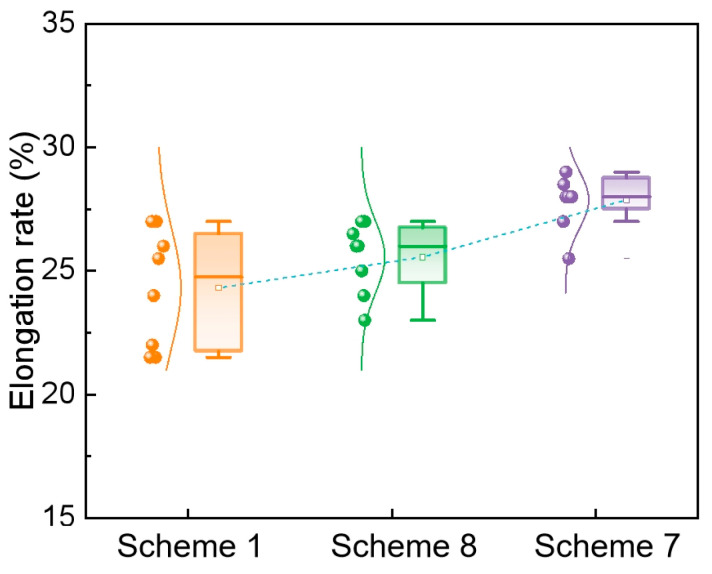
Elongation distribution of rolled plate.

The elongation of the specimens was also measured. The average elongation values were 24.3% for Scheme 1 (SR only), 25.6% for Scheme 8 (EMS only), and 27.7% for Scheme 7 (EMS + SR), as presented in Figure 13. A clear inverse correlation is observed: a lower slab centerline segregation index corresponds to a higher elongation in the rolled plate. Simultaneously, the fluctuation in elongation (as indicated by the standard deviation) decreased significantly as the slab segregation index decreased. This improvement is due to the more uniform composition resulting from lower segregation, which in turn leads to a more homogeneous microstructure. A homogeneous microstructure experiences less localized stress concentration during deformation, facilitating more uniform plastic flow. This manifests as both the higher average elongation and the reduced scatter in the elongation data. Therefore, compared to the other schemes, Scheme 7 (EMS + SR) yields a rolled plate with the most optimal combination of ductility and consistent tensile properties. This underscores the importance of minimizing slab centerline segregation to achieve superior and uniform product performance.

The uniformity of mechanical properties of rolled plate is characterized by the standard deviation of tensile strength and the standard deviation of elongation. The mechanical properties of rolled materials under different scheme conditions are analyzed. When the slab center segregation index in scheme 1, scheme 8, and scheme 7 are 1.23, 1.20 and 1.15, respectively, the tensile strength deviations are 12.15 MPa, 9.81 MPa and 8.03 MPa, and the standard deviations of elongation were 2.4%, 1.5% and 1.1% respectively. Therefore, the standard deviations of tensile strength and elongation of Scheme 7 are smaller than those of the other two schemes, and its mechanical properties are more uniform. In the industrial production of E355 steel, the slab center segregation is reduced by optimizing the parameters of EMS and SR. The deviations of tensile strength and elongation rate are reduced, which is beneficial for the production of high-quality steel. From An’s work [16], the macrosegregation in bloom can be effectively reduced to 0.96∼1.08 with the electromagnetic stirring and soft reduction applied. Accordingly, the grade of network and banded carbide could be significantly reduced to 1. Therefore, improving the macrosegregation of the strand can reduce the banded structure defects in the rolled plate.

The uniformity of the mechanical properties of the rolled plate was quantified using the standard deviations of both tensile strength and elongation. The mechanical properties of the rolled materials produced under the different scheme conditions were analyzed accordingly. The corresponding data are summarized as follows: for Scheme 1 (centerline segregation index = 1.23), the standard deviations of tensile strength and elongation were 12.15 MPa and 2.4%; for Scheme 8 (1.20), they were 9.81 MPa and 1.5%; and for Scheme 7 (1.15), they were 8.03 MPa and 1.1%. Consequently, Scheme 7 exhibits the smallest standard deviations for both tensile strength and elongation, indicating the most uniform mechanical properties among the three schemes.

This study demonstrates that in the industrial production of E355 steel, slab centerline segregation can be effectively reduced by optimizing EMS and SR parameters. This leads to a reduction in the deviations of tensile strength and elongation, which is highly beneficial for the production of high-quality steel. This finding is consistent with the work of An et al. [16], who reported that macrosegregation in blooms could be reduced to a range of 0.96–1.08 through the application of electromagnetic stirring and soft reduction. In their work, the severity grade of carbide networks and banding was correspondingly reduced to as low as 1.0. Therefore, mitigating macrosegregation in the strand is a critical and effective strategy for minimizing banded structure defects in the final rolled product.

## 4. Conclusions

(1) In the continuous stirring mode, increasing the stirring current enhances the equiaxed crystal ratio and reduces centerline segregation, with the effect saturating beyond 240 A. The alternating stirring mode is more effective in fragmenting columnar dendrites and promoting solute homogenization, leading to a higher equiaxed zone ratio and lower centerline segregation. EMS and SR combined application yields the optimal improvement in slab internal quality, reducing the centerline segregation index to 1.15.
(2) Centerline segregation in the slab is inherited in the rolled plate, manifesting as banded structures. More severe slab segregation results in more pronounced banded structures. Mitigating slab segregation significantly improves the uniformity of the mechanical properties of the rolled plate. As the centerline segregation index decreased from 1.23 to 1.15, the standard deviations of tensile strength and elongation decreased from 12.15 MPa and 2.4% to 8.03 MPa and 1.1%, respectively, indicating reduced property scatter and enhanced homogeneity. Optimizing the parameters of EMS and SR is a critical and effective strategy for controlling macrosegregation in continuous casting strands. This is essential for minimizing banded structure defects and achieving superior, uniform mechanical properties in high-quality steel plates.

## Figures and Tables

**Table 1:** EMS and SR parameters.

Scheme	Stirring Mode	Current (A)	Reduction Zone (m)	Reduction Amount (mm)
1	-	0	17~21	3.0
2	Continuous	160	17~21	3.0
3	Continuous	240	17~21	3.0
4	Continuous	320	17~21	3.0
5	Alternative	160	17~21	3.0
6	Alternative	240	17~21	3.0
7	Alternative	320	17~21	3.0
8	Alternative	320	-	-

**Table 2:** Banded structure in the plate and center segregation in the slab.

Scheme	Working Condition	Band Grade	Center Segregation Index
1	Only SR	5.0	1.23
8	Only EMS	3.0	1.20
7	Both with SR and EMS	2.0	1.15

## Data Availability

The original contributions presented in this study are included in the article. Further inquiries can be directed to the corresponding author.
